# Dataset on the shooting and rooting ability of *Morus alba* using waste tea residue derived carbon dots as an alternative of growth plant stimulator

**DOI:** 10.1016/j.dib.2020.105345

**Published:** 2020-02-28

**Authors:** Ravindra D. Waghmare, Anil H. Gore, P.V. Anbhule, Daewon Sohn, Govind B. Kolekar

**Affiliations:** a Department of Agrochemicals and Pest Management, Shivaji University, Kolhapur, 416004, Maharashtra, India; b Department of Chemistry, Uka Tarsadia University, Gopal Vidyanagar, Barodli, 394350, Gujarat, India; c Fluorescence Spectroscopy Research Laboratory, Department of Chemistry, Shivaji University, Kolhapur, 416004, Maharashtra, India; d Department of Chemistry, Hanyang University, Seoul, 04763, South Korea

**Keywords:** WTR-CDs, Plant propagation, Nodal explant, *ex vitro*

## Abstract

The data article *ex vitro* (vegetative plant propagation) culture techniques are sustainable alternatives to the large-scale production of economically important plant species. *Morus alba* is an essential species that is mainly considered to be economically important due to their potential use as silk production, medicine and food. In this work, we evaluated the data of effects of different concentration of Waste Tea Residue Carbon Dots (WTR-CDs) on the ex vitro growth of morus. This dataset can be beneficial for researchers finding alternative eco-friendly, biodegradable and cost-friendly substitute for plant growth stimulator that are helpful for plant propagation during plant production program. Time consuming and low germination ratio of seeds are the most restricting triggers for commercial use for large-scale cultivation of plant species. Use of WTR-CDs in *ex vitro* culture technology is an appropriate alternative approach for large-scale production of plants within a short period of time.

**Table:** Specifications Table

Subject	Agronomy
Specific subject area	Plant propagation or ex vitro.
Type of data	Table Graph Figure
How data were acquired	An experimental examination based on the number of bud formation, growth of bud, length of bud, time (days), root formation, length of root and concentration of WTR-CDs
Data format	Analysed
Parameters for data collection	Propagated in ex vitro condition, effect of concentration of WTR-CDs, measurement of bud (shoot) and root growth, observation of total roots number.
Description of data collection	Based on total observation and measurement of parameters such as time (days), growth factors and effect of different concentration of WTR-CDs on plant propagation.
Data source location	Shivaji University Kolhapur, Maharashtra, India. latitude 16.6780° N, Longitude 74.2555° E
Data accessibility	The data are available within this article

**Table:** 

**Value of the Data** • This dataset displayed that the shooting and rooting ability of WTR-CDs was successful with the application of alternative plant growth stimulator in *ex vitro* plant propagation. • Use of WTR-CDs in *ex vitro* culture technology is an appropriate alternative approach for large-scale production of plants within a short period of time. • Examined dataset can be beneficial for finding alternative eco-friendly, biodegradable and cost-friendly substitute for plant growth stimulator.

## 1 Data description

**Fig. 1:**
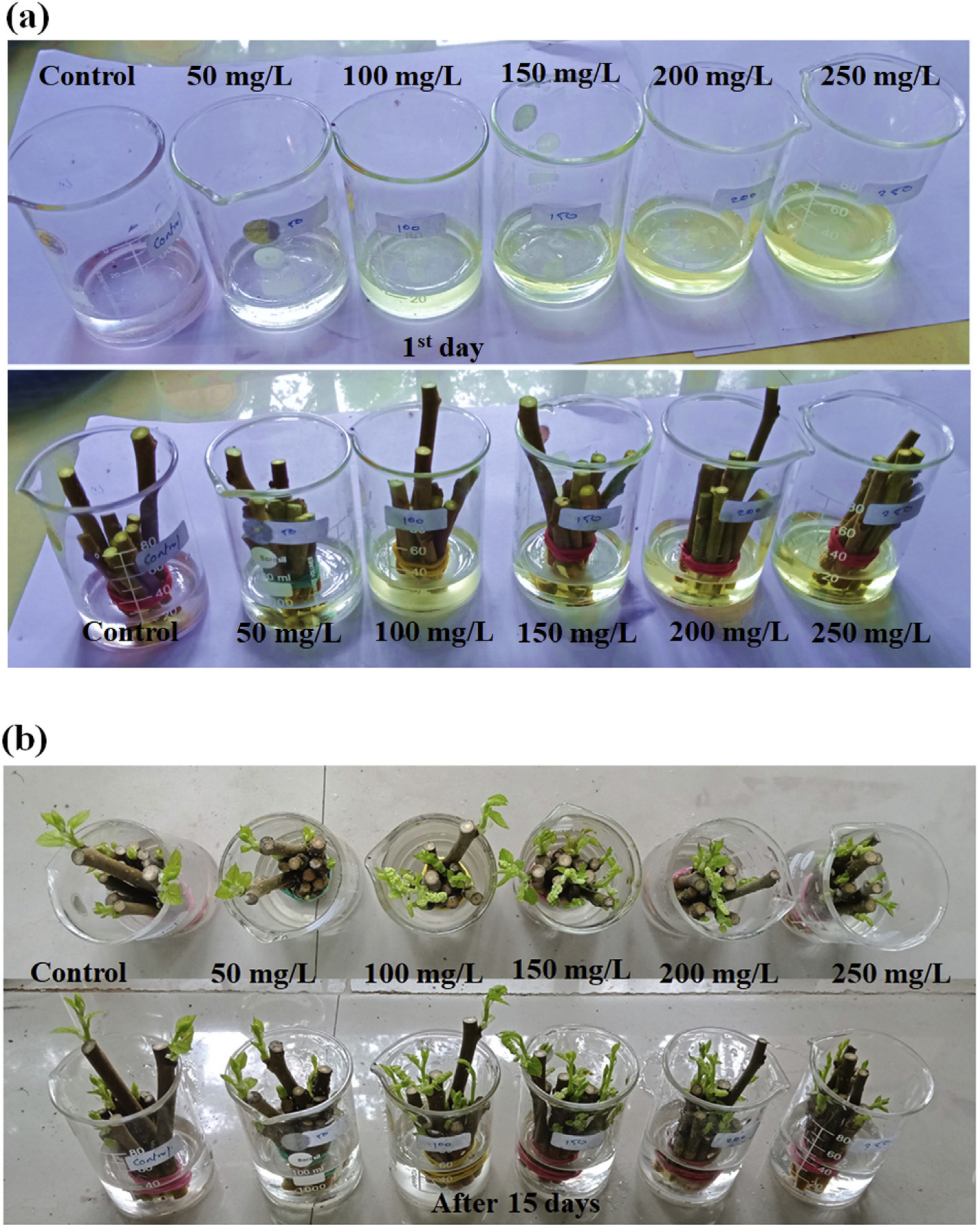
(a) Treatment of different concentration of WTR-CDs and (b) Effect of different concentration of WTR-CDs on bud growth. (n = 10).

**Fig. 2:**
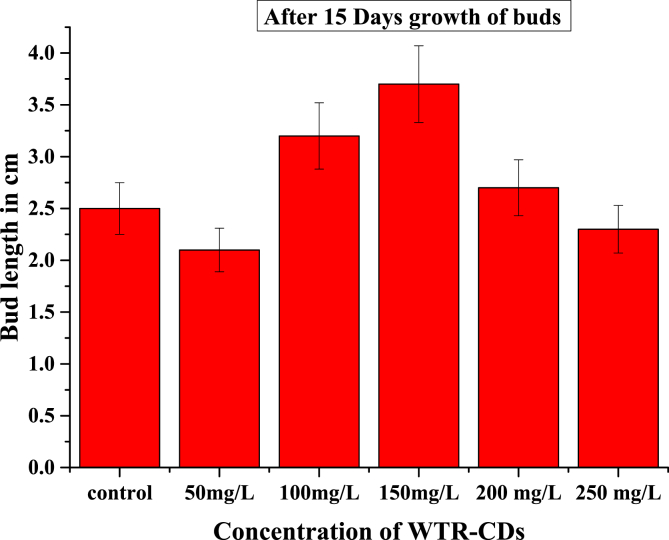
Effect of different concentration of WTR-CDs on bud growth and number of bud formation. (n = 10).

**Fig. 3:**
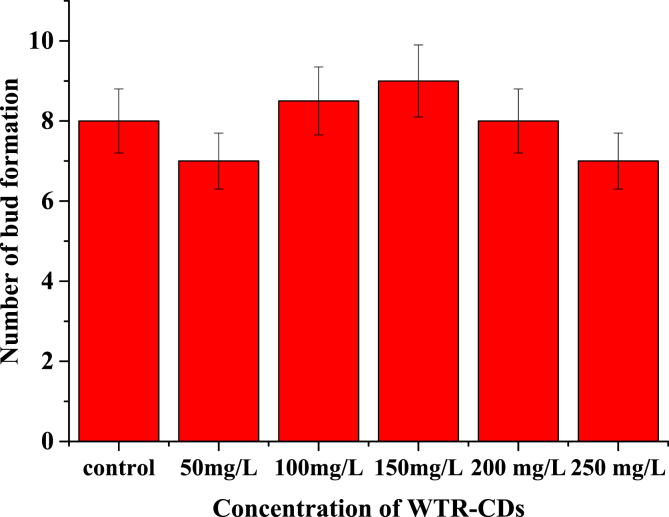
Effect of different concentration of WTR-CDs on number of bud formation (After 15 days) (n = 10).

**Fig. 4:**
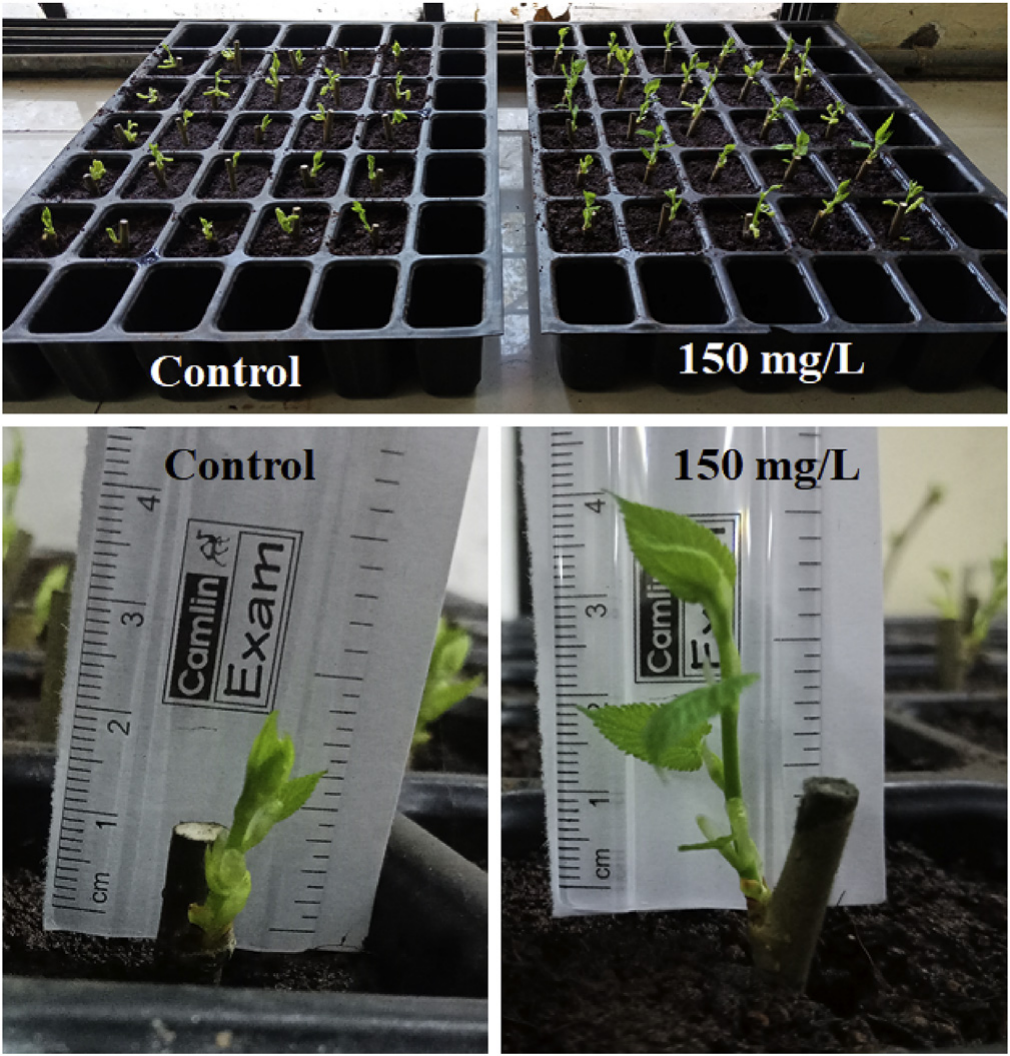
Effect of optimized concentration of WTR-CDs on bud formation (After 15 days) (n = 25).

**Fig. 5:**
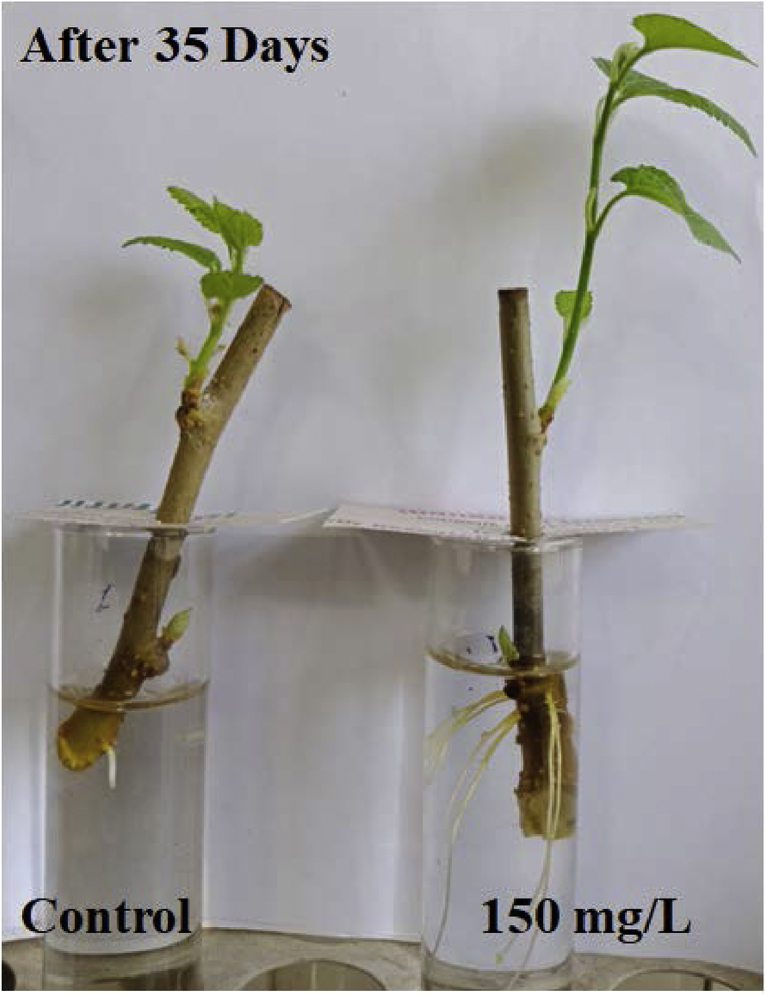
Effect of optimized concentration of WTR-CDs on rooting (After 35 days).

**Fig. 6:**
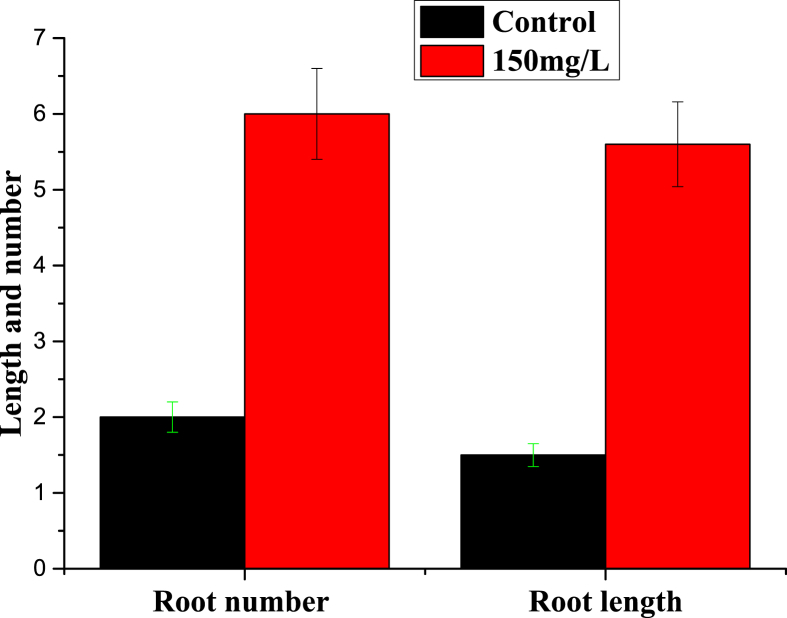
Effect of optimized concentration of WTR-CDs on rooting (After 35 days).

Dataset in figures (Fig. 1, Fig. 2, Fig. 3, Fig. 4, Fig. 5) display the effect of WTR-CDs treatment on number of sterilized nodal explant shoot regeneration of *M. alba* nodal explant after 35 days of Propagation. In Fig. 1, Fig. 2, Fig. 3 the different concentration effect of WTR-CDs on number of bud formation and growth of bud was displayed. From this Fig. 1, Fig. 2, Fig. 3 it is seen that the lowest level of bud expression in explant nodal growth at the control, 50, 100, 200 and 250 mg/L treatment while it is the fastest and significant growth rate of explants treated with 150 mg/L. At the high concentration of WTR-CDs shows negative effect on shoot formation [2]. Fig. 4 showed the comparison between control and optimized concentration of WTR-CDs as well as statistical analysis data which shown in Table 1. Fig. 5, Fig. 6 discovered the shoot growth length, number of roots and root length of nodal explant of *M. alba*.

**Table 1:** Effect of optimized concentration of WTR-CDs on bud growth of *M. alba* (Day 15).

Anova: Single Factor			
SUMMARY			
Groups	Count	Sum	Average
0 (Control)	25	55.8	2.232
150 mg/L	25	98.5	3.94

## 2 Experimental design, materials, and methods

### 2.1 Materials and methods

Mulberry plants (*M. alba*) were taken from a single mother plant in the Shivaji University, Kolhapur (Maharashtra) campus. Around 60 healthy nodal explants cutting in the form of pencil size diameter and 5–6 cm length for plant propagation. Those nodal explants were cut near about 45° angel from the base side of plant stick stem with one bud, Instead, 0.1% (w/v) mercury chloride was used for surface sterilized for 30 min after that treatment rinsing them with sterile distilled water for five times.

### 2.2 Preparation and characterzation of WTR-CDs

The synthesis and characterization of WTR-CDs is discussed in the article: ‘Sustainable carbon nanodots synthesized from kitchen derived waste tea residue for highly selective fluorimetric recognition of free chlorine in acidic water: A waste utilization approach’ https://doi.org/10.1016/j.jtice.2018.10.014 [1].

### 2.3 WTR-CDs treatment

Sterilized nodal explant of *M. alba* were equally distributed (10 per set) in six set, set II, III, IV, V and VI was dipped in 20 mL of 50, 100, 150, 200 and 250 mg/L WTR-CDs solutions respectively for 7 days in beaker (Fig. 1 a). Similarly another set (I) was dipped in the water (control).

### 2.4 Optimized concentration treatment of WTR-CDs

The plan of the experimentation was a Randomized Complete Block Design (RCBD) where five cuttings of nodal explant were exposed to two treatments (control and optimized concentration of WTR-CDs) and five times replicate. After bud formation, some bud formed *M. alba* explant samples were subjected to the plantation in the plastic trays. One set was kept in test tube under observation to check the root formation. The following observations data were given at the end of the experiment; Number of bud formation, growth of bud, number of roots and root length of explant.

### 2.5 Statistical analysis

The dataset collected was examined using Analysis of Variance (Single factor ANOVA) and the means were using *t*-Test: Paired Two Sample for Means.

## Contribution

Conceptualization, R.D.W., A.H.G. and G.B.K.; Methodology, R.D.W. and A.H.G.; Data curation, R.D.W.; Writing-original draft preparation, R.D.W. and A.H.G; Writing-review & editing, P.V.A., D.S., and G.B.K.; Visualization, R.D.W. and A.H.G.; Supervision, P.V.A. and G.B.K.
