# Supplementary material for: Dataset on the shooting and rooting ability of Morus alba using waste tea residue derived carbon dots as an alternative of growth plant stimulator
Source: Data Brief. 2020 Feb 28;29:105345. doi: 10.1016/j.dib.2020.105345 (PMC7062930; doi:10.1016/j.dib.2020.105345)
Supplement: Multimedia component 1 [file mmc1.docx]

**Supplementary information**

**Article Title**

Dataset on the shooting and rooting ability of Morus S. using waste tea residue derived carbon dots as an alternative of growth plant stimulator.

**Authors**

Ravindra D. Waghmare,^1,3^ Anil H. Gore,^2^ P. V. Anbhule,^1,3^ Daewon Sohn,∗^4^ Govind B. Kolekar^∗1, 3, 4^

**Affiliations**

^1^Department of Agrochemicals and Pest Management, Shivaji University, Kolhapur-416004 Maharashtra, India

^2^Department of Chemistry, Uka Tarsadia University, Gopal Vidyanagar, Barodli-394350, Gujarat

^3^Fluorescence Spectroscopy Research Laboratory, Department of Chemistry, Shivaji University, Kolhapur-416004 Maharashtra, India

^4^Department of Chemistry, Hanyang University, Seoul 04763, South Korea.

***Corresponding Author:** Prof. Govind B. Kolekar (gbkolekar@yahoo.co.in),

Prof Daewon Sohn (dsohn@hanyang.ac.kr)

Tel: +91-231-2609391, Fax: +91-231-2692333

**Synthesis and characterizations of WTR-CDs is present in this published article**

Sustainable carbon nanodots synthesised from kitchen derived waste tea residue for highly selective fluorimetric recognition of free chlorine in acidic water: A waste utilization approach

https://doi.org/10.1016/j.jtice.2018.10.014
